# Determination *in vivo* viability of a transfused platelet product by corrected count increment and percentage platelet response

**DOI:** 10.11604/pamj.2017.27.226.12116

**Published:** 2017-07-28

**Authors:** Nancy Wanjiru Thuku, Kibet Shikuku, Amos Mbugua

**Affiliations:** 1 MSc student at Jomo Kenyatta University of Agriculture and Technology, Head of Haematology and Blood Transfusion Science Section at Mbagathi District Hospital, Nairobi, Kenya; 2 Lecturer at University of Nairobi, College of Health Sciences Department of Human Pathology, Haematology and Blood Transfusion Unit, Nairobi, Kenya; 3 Lecturer at Jomo Kenyatta University of Agriculture and Technology College of Health Sciences, Nairobi, Kenya

**Keywords:** Platelet, Refractoriness, immune

## Abstract

**Introduction:**

For many years, platelet concentrates have been used for the prevention as well as treatment of bleeding disorders, especially in those patients with haematological problems involving platelet disorders as well as refractoriness, In addition, platelet concentrates (PCs) have been widely used to support patients undergoing bone marrow transplantation or who are receiving myelotoxic treatments. The aim of this study was to determine the quality of platelet concentrates by assessing platelet counts, volume, pH changes, swirling, residue of the red blood cells and white blood cell counts. Assess the in vivo viability of a transfused platelet product using the corrected count increment (CCI) and the percentage platelets response (PPR). This descriptive analysis study was done in Kenyatta National Hospital Blood Transfusion Unit between July 2016 and December 2016.

**Methods:**

The in vitro Platelets concentrates quality was accurately determined and assessed using certain parameters. Platelet concentrates in ethylene diamine tetra acetic acid (EDTA) was used for analysis using Cell-Dyn 3700 analyser. The volume of PCs used was an average of 2mls of PCs, the pH was measured using digitalised Hanna edge pH kit. Agitation was done using Helmer agitator and centrifugation was done using Roto silenta 630 RS centrifuge. The *in vivo* viability of a transfused product was determined using corrected count increment (CCI) and percentage recovery (PR) between 1 and 20-hour after transfusion. Pre and post-transfused whole blood in EDTA collected from the recipients was analysed to access the functional platelets in the circulation. Data analysis was done using SPSS.

**Results:**

A total of 384 platelet concentrates were analysed and used in transfusion. The majority 96, (40%) were O Rhesus D+ and the least being AB Rhesus D-at (1%).Centrifugation, separation and agitation was done according to standard procedure (n=384). Only (246 (65%) of the concentrates were found fit for use out of a total of (n=384) leaving 138 (35%) which did not meet the KNH/KNBTS criteria. The minimum specifications for platelet count are 5.5 x (10^9^). The duration of 3 days of storage on average, the WBC count (10^9^) was Mean ± SD 4.50 ± 3.50. Using the Hanna edge pH kit the pH Mean was ±SD 7.18 ± 8.82 and the used Volume (Mls) was at 55 ± 15. The concentrate was issued within 3 days of processing. After transfusion, the percentage platelet response (PPR) was 72% in male recipients at 1-hour and 30% at 20-hours while 69% in female recipients at 1-hour and 25% at 20-hours. The invivo viability of platelet product had a corrected count increment (CCI) of 75% ≥ 7500 at 1-hour and CCI of18% ≥ 30% at 20-hours in male recipients. In the same study, the female recipients had a CCI of 80% ≥ 7500 at 1-hour and a CCI of 25% ≥ 30% at 20-hours.

**Conclusion:**

The findings on platelets concentrates quality 65% met platelets transfusion criteria while 35% did not. On preparation of platelets concentrates there was high counts of white blood cells 4.5±3.5×10^9^than recommended counts by Kenya National Blood Transfusion Services < 0.83×10^9^. Both percentage platelet response (PPR) and corrected count increment (CCI) were very low at 20 hours compared to British committee for standards haematology criteria for successful increment of platelet products (PPR ≥ 30% and CCI ≥ 7500). Apheresis platelets transfusion can be introduced at KNH and use of leukoreduction performed on the platelet concentrates which are prepared within the Hospital. With such rate of refractoriness, additional tests to confirm the real cause of unviability of platelets in the patients need to be performed. Recipients should be done evaluation of the pattern of refractoriness followed by HLA compatibility testing. In addition, if there is a high, compatible cross-matched, selected apheresis platelet concentrate pint should be transfused. This unviability was due to recipients with either immune-mediated refractoriness or non-immune mediated refractoriness.

## Introduction

Platelets concentrates are mainly used in thrombocytopenic patients mostly commonly as a result of myelo-supplessive therapy. Increased consumption as in disseminated intravascular coagulopathy (DIC), a plastic anaemia due to qualitative defects in Bernard Solier syndrome or in quantitative defects in decreased platelets counts which is seen in myeleosuppressive drugs like chemotherapy [1]. A normal platelets count ranges from 150,000-450,000 platelets per microliter of blood and values outside this range do not indicate disease. Platelets that need emergency treatment are a count below 50,000 per microliter [2] but at Kenyatta National Hospital, majority of recipients are considered emergency transfusion of platelet concentrates at a count of 20×109 platelets in the circulation. Thrombocytopenia correlates with bleeding complications, guidelines advise to institute a platelet transfusion policy to prevent and treat bleeding complications.Studies concerning platelet concentrate transfusions conducted between the early eighties until the late nineties mainly investigated product modifications to reduce adverse reactions, such as (non)-immunological refractoriness and febrile transfusion reactions. It was only in the last decade that clinical efficacy became an issue, and more to blood bankers than to clinicians [3].

Modifications have been performed to improve the safety profile are typically characterised by removing one or more components after blood donation. With regard to efficacy as an endpoint for platelet transfusion in recent years have shown increasing doubts and problems with the commonly used endpoints: corrected count increments and bleeding. The corrected count increment (CCI) is a ratio correcting for a measure of blood volume and the number of transfused platelets. This method has been challenged by several authors in the field with as main arguments a bias in favour of a preparation technique with fewer platelets combined without adequately estimated blood volume and doubts regarding the usefulness of the CCI as a surrogate outcome measure as it does not predict the clinical outcome bleeding [4]. The methods for evaluation of in vivo viability (therapeutic efficacy) of platelets are corrected count increment and percentage platelet recovery (PPR) [5]. There is a constant demand of platelet concentrates in KNH, as a referral Hospital, in the management of different conditions. While platelet concentrates are highly perishable, costly and needs a thorough investigation in the point of collection, separation, storage, and selecting compatible recipient, administration requiring maximum quality efficiency and safety. This study sought to investigate the *in vivo* viability of a transfused platelet product. The objective of this study was therefore to determine *in vivo* viability of a transfused platelet product by corrected count increment and percentage platelet response.

## Methods

The study was conducted in Kenyatta National Hospital Blood Transfusion Unit. In addition preparation of platelet concentrates was done at Kenya National Blood Transfusion Services which is beside Kenyatta National Hospital. This was a descriptive analysis study in determination of platelet counts in platelet concentrates and effectiveness of platelets after transfusion at Kenyatta National Hospital Blood Transfusion Unit. A total of 384 platelet concentrates for assessment of quality of platelet concentrates was included after fulfilling the inclusion criteria which was systematically selected [6]. During study period, 123 recipients of the platelet concentrates (aged between 1-60 years) was recruited for determination of *in vivo* viability of transfused platelet products. The study was conducted out between July and December 2016. The sample size for the study was calculated using the formula; n=Z^2^P (1-P)/d^2^ [7]. The sample size calculated was 246. The sampling method was systemic sampling which was quick and easy way to obtain unbiased data and is less expensive

**Corrected count increment**: CCI: apheresis platelet concentrates = (Post-platelet count-Pre-platelet count)(BSA) platelets transfused (x10^9^) CCI >Body surface area (M2) Example: a woman transfused with SDP (4 x 10^9^). Platelet count pre=8, post=42. BSA=1.5 M^2^

**Percentage Platelet Recovery (PPR)**: Percent Predicted Count (PPC) =No. platelets transfused x 0.67 x .001 blood volume (mL) Percent Platelet Recovery (PPR) = (post-platelet - pre-platelet count)x 100% percent predicted count Permission of carrying out research was acquired from the KNH-UON ERC. In addition written informed consent/assent was obtained from parents/guardians willing to participate by the clinician following a detailed explanation of the study to them. In case of an illiterate parent/guardian, a literate witness who did not have any connection in the study, signed. It is only after the patient/parent/guardian had signed the consent/assent did the study began.

**Laboratory procedures**: Two milliters of whole blood was collected following sample collection guidelines as described by Kenyatta Hospital Haematology Laboratory standard operating procedure (S0P). The samples were preserved using ethylene diamine tetra acetic acid EDTA and transported to Haematology Laboratory for analysis. Laboratory analysis was done in Haematology Laboratory, Kenyatta National Hospital. The standard operating procedure (SOP) for preparation of platelet concentrates as described by KNBTS was used. The in vivo viability of platelets product after transfusion was determined by the percentage of the transfused platelets recovered in the recipient's circulation immediately after transfusion (% recovery) and by the life span in circulation of these recovered platelets (survival). Two methods were used in this study for platelet survival. These are corrected count increment (CCI) and percentage platelet response (PPR). Pre and post blood from recipients who have been transfused with platelet concentrates were analysed and both corrected count increment and percentage recovery (PPR) were calculated after 1-hour and 20-hours. This was evaluating platelet transfusion response. The measurements were calculated as follows: CCI= (Post-platelet count-Pre-platelet count) (BSA (Body Surface Area) in M^2^) /platelet transfused ×10^9^ Normal values ≥ 7500 at 1 hour and ≥ 5000 at 20 hours. PPR = (post platelet counts-pre platelet counts) ×100%/percentage response counts Normal values of PPR is ≥ 60% at 1-hour and ≥ 30% at 20-hours. Quality control was ascertained using AcT Cell Control Plus from Phillips Healthcare Technologies Limited (these are commercial cells control).

**Data management and analysis**: All collected data were entered into computer database in Microsoft excel (Ms-excel) computer application. To avoid loss, back up of the data was burned on compact discs, external hard disc and pen drives (flash discs). Validation and cleaning of the data was done during data collection and entry and later exported to and analysed by Statistical Package for Social Sciences (SPSS) version 20. Data analysis was conducted using descriptive, Statistical Package for Social Sciences, (SPSS) software. After being entered into MS-EXCEL, the number and percentage distribution was calculated. The number and percentage distribution was calculated and the student t-test was used to test significance at P ≤ 0.05. Descriptive means, frequencies and percentages was used to describe and summarizes data. Tables, pie charts and graphs were used to present results.

## Results

Of the transfused patients 72% were female while 28% were males. After platelets concentrates quality parameters assessment only 65% (246) were fit for platelets transfusion and 35% were unacceptable (Table 1). In this study the majority of patients transfused with platelets concentrates 75% male and 80% female, after calculating their percentage platelets response were ≥ 60% at one hour. After 20 hrs it's only a few of them 18% male and 25% female had PPR ≥ 30% indicating refractoriness on the majority of patients. The causal agent of refractoriness could be immune or non-immune. In this study the evaluation of platelet transfusion was done using corrected count increment (CCI). This is in vivo measurement platelet survival which requires a 1-24 hour post-platelet count. In addition, the 1-hour post transfusion CCI was 72% ≥ 7500 in male recipients while female recipients had 69% ≥ 7500 which was normal but after 20-hour post transfusion the CCI was 30% ≥ 5000 in male recipients while 25% ≥ 5000 was seen in female recipients. This scenario was not a successful transfusion increment and was an indication of refractoriness on the majority of recipients (Table 2).

**Table 1:** Quality parameters of platelet concentrate

	Platelets count 10^9^	Duration of storage in days	WBC Count 10^9^	RBC Residue count 10^9^	PH of the platelet concentrate	Volume (mls) of PCs
PCs Met the criteria	>5.5	2.2±0.8	4.5±3.5	7.18±8.82	6.2±0.2	55±25
PCs Count (%)	246 (65)	384 (100)	77 (20)	Not specified	372(97)	376(98)
PCs did not meet criteria	<5.0	2.1±0.9	3.1±2.8	2.90±3.3	7.3±0.5	7±5.6
PCs Counts (%)	138(35)	-	307(80)	Not specified	12(3)	8 (2)
Total PCs	384	384	384	384	384	384

Out of the 384 PC pints used in this study and based on the research objective one, there were 246 (65%) pints which met quality PCs counts. The other 138 (35%) pints did not meet the criteria for PCs count as stipulated by the National Blood Transfusion Services criterion from the platelet concentrates. The proceeding analysis was based on the sample of 246 pints

WBCs –White blood cells

RBCs - Red Blood Cells

PH – Potential of Hydrogen

mls – Millilitres

**Table 2:** The in vivo platelets response

N=123	CCI	CCI	CCI	CCI	PPR	PPR	PPR	PPR
Time after Transfusion Of PCs	1hr	20hrs	1hr	20hrs	1hr	20hrs	1hr	20hr
Viability of PCs	≥7500	≥5000	≤7500	≤500	≥60%	≥30%	≤60%	≤30%
Male	72%	30%	28%	70%	75%	18%	25%	82%
Female	69%	25%	31%	75%	80%	25%	20%	75%

The in vivo platelets response. The platelets which met acceptable criteria were transfused to patients on requisition and their Corrected Count Increment (CCI) and Percentage platelet Response (PPR) calculated

There is increase of none viable platelets of 20-hour after transfusion

CCI -corrected count increment.

PPR -percentage platelet response

PCs -Platelet concentrates

N-number of recipients

## Discussion

**Figure 1:**
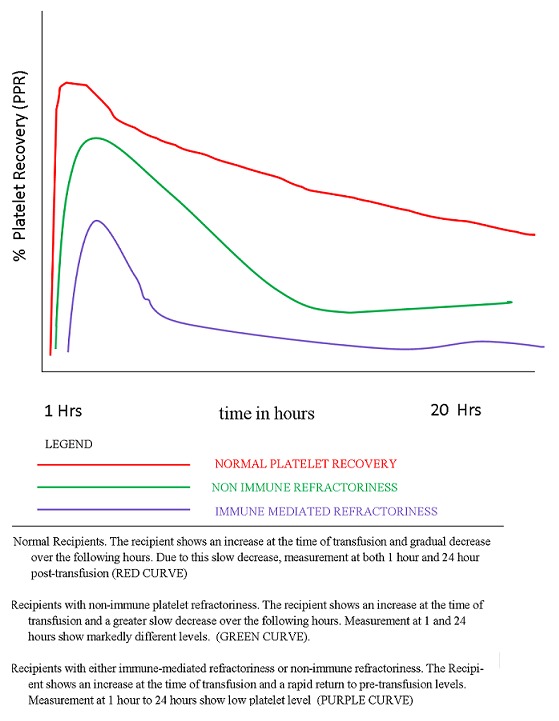
Platelet in normal and refractoriness recipients

The in vivo viability of the platelet products was done from the calculation as corrected count increment (CCI) and in this study, 72% of male recipients showed a CCI ≥ 7500 after 1-hour transfusion and 30% CCI≥ 5000 after 20-hour while the 69% of the female recipients had a CCI ≥ 7500 after 1-hour and 25% had a CCI ≥ 5000 after 20-hour. After transfusion of platelets concentrates on patients, 75% male and 80% female had percentage platelets response › 60% but after 20 hrs only 18% of male and 25% of female had ≥ 30% PPR indicating low viability of transfused platelets products (Figure 1). This reflects a very low viability of platelet product according to British committee for standards in haematology criteria resulting to successful transfusion for CCI. Failure of the majority of these recipients to achieve the expected platelet increment after 20-hours could be due to alloimmunization to HLA class 1 antigen or HPA to HLA class 1 antigen or HPA.

## Conclusion

The findings of this study suggest apheresis platelets transfusion need to be introduced at KNH and use of leukoreduction performed on the platelet concentrates which are prepared within the Hospital. With such rate of refractoriness, additional tests to confirm the real cause of unviability of platelets in the patients need to be performed. These tests include bone marrow examination, polymerase chain reaction, immunohistochemistry and antibody screening test for alloimmunization, passive immunity and auto antibodies. Antibody elusion tests must be included for confirmation antibodies. In the process of separation of platelet product (opts press release), one should be carefully not to allow White blood cells which result to HLA antibodies to recipient circulation after transfusion. KNH should ensure those recipients with low profile of viability of platelet counts to perform evaluation of pattern of refractoriness using 1-hour and 20-hour pre and post transfusion levels of platelets viability, perform cross- match compatibility and HLA- compatibility testing and last but not least to recommend future report of usage of transfused platelets concentrates to these recipients.

### What is known about this topic

- A recipient has refractoriness to platelet transfusions if the circulating platelet levels fail to increase by at least 10,000/microliter after transfusion of an appropriate dose of platelets. There are multiple causes of platelet refractoriness, both immune-mediated and non-immune-mediated;
- Immune-mediated refractoriness is due to antibodies made by the patient that recognize an epitope on the transfused platelets, most commonly human leukocyte antigen (HLA) class I;
- Non-immune-mediated refractoriness is due to a process that significantly decreases the circulation time of transfused platelets, non-immune causes include splenomegaly, fever and infections.

### What this study adds

- Assessed the in vitro quality of platelet concentrates by measuring the following parameters: volume, swirling, pH changes, platelets count, residue of red blood cells and white blood cells;
- Determined in vivo viability of a transfused platelet product by corrected count increment and percentage platelet response;
- The study adds the importance of performance of cross- matching platelets concentrates (PCs), HLA testing and evaluation of future usage of PCs before transfusion is done to refractoriness patients.

## Competing interests

The authors declare no competing interest.
